# Maximal parathyroid gland diameter as a predictive factor for autograft-dependent recurrent secondary hyperparathyroidism after total parathyroidectomy

**DOI:** 10.3389/fendo.2023.1175237

**Published:** 2023-06-15

**Authors:** Takahisa Hiramitsu, Yuki Hasegawa, Kenta Futamura, Manabu Okada, Norihiko Goto, Shunji Narumi, Yoshihiko Watarai, Yoshihiro Tominaga, Toshihiro Ichimori

**Affiliations:** Department of Transplant and Endocrine Surgery, Japanese Red Cross Aichi Medical Center Nagoya Daini Hospital, Nagoya, Japan

**Keywords:** secondary hyperparathyroidism, parathyroidectomy, recurrent secondary hyperparathyroidism, autograft, parathyroid gland

## Abstract

**Introduction:**

Following total parathyroidectomy (PTx), transcervical thymectomy, and forearm autograft for secondary hyperparathyroidism (SHPT), recurrent SHPT can occur in the autografted forearm. However, few studies have investigated the factors contributing to re-PTx due to autograft-dependent recurrent SHPT before the completion of the initial PTx.

**Methods:**

A total of 770 patients who had autografted parathyroid fragments derived from only one of the resected parathyroid glands (PTGs) and who had undergone successful initial total PTx and transcervical thymectomy—defined by serum intact parathyroid hormone level < 60 pg/mL on postoperative day 1—between January 2001 and December 2022 were included in this retrospective cohort study. Factors contributing to re-PTx due to graft-dependent recurrent SHPT before the completion of the initial PTx were investigated using multivariate Cox regression analysis. Receiver operating characteristic (ROC) curve analysis was performed to obtain the optimal maximum diameter of PTG for autograft.

**Results:**

Univariate analysis showed that dialysis vintage and maximum diameter and weight of the PTG for autograft were significant factors contributing to graft-dependent recurrent SHPT. However, multivariate analysis revealed that dialysis vintage (*P*=0.010; hazard ratio [HR], 0.995; 95% confidence interval [CI], 0.992–0.999) and the maximum diameter of the PTG for autograft (*P=*0.046; HR, 1.107; 95% CI, 1.002–1.224) significantly contributed to graft-dependent recurrent SHPT. ROC curve analysis showed that < 14 mm was the optimal maximum diameter of PTG for autograft (area under the curve, 0.628; 95% CI, 0.551–0.705).

**Conclusions:**

The dialysis vintage and maximum diameter of PTG for autograft may contribute to re-PTx due to autograft-dependent recurrent SHPT, which can be prevented by using PTGs with a maximum diameter of < 14 mm for autograft.

## 1 Introduction

Patients with prolonged chronic kidney disease often experience secondary hyperparathyroidism (SHPT) (1). Calcimimetics can be a treatment of choice for managing SHPT (2). However, some patients are refractory to medical treatment or cannot take calcimimetics. Consequently, parathyroidectomy (PTx) is the final treatment option for these patients. There are various surgical procedures for treating SHPT, including subtotal PTx that involves removing 3.5 parathyroid glands (PTGs) while leaving 40–80 mg of PTG tissue in the neck to prevent hypoparathyroidism. Autografting is not necessary during subtotal PTx, and previous reports have shown that similar clinical outcomes can be obtained with subtotal PTx as with total PTx and autograft (3, 4). However, there is a potential risk of dissemination and recurrent SHPT due to the remaining PTG, which is a concern (5). Subtotal PTx is recommended for patients who anticipate kidney transplantation in the near future (6, 7). As improved kidney function does not stimulate the remaining PTG, it can prevent recurrent SHPT. However, for patients who require long-term dialysis or anticipate kidney transplantation, total PTx, transcervical thymectomy, and forearm autograft are recommended (6). These surgical procedures can prevent persistent or recurrent SHPT in the neck area, and any recurrent cases of SHPT owing to autografted PTGs in the forearm can be easily removed under local anesthesia (6). Total PTx, transcervical thymectomy, and forearm autograft are commonly performed (8). Previous reports have demonstrated the efficacy of PTx in improving several clinical outcomes, such as bone density, osteodystrophy, cardiovascular events, and mortality (1, 9–12). However, persistent or recurrent SHPT, which can occur in the neck or mediastinal area and in the autografted forearm after the initial PTx, remains a serious problem, resulting in re-PTx (13, 14). Previous studies have often investigated persistent or recurrent SHPT in the neck or mediastinal area following initial PTx (14, 15). Remnant PTGs in the neck or mediastinal area after initial PTx are stimulated under chronic kidney disease conditions, leading to the causative PTGs for persistent or recurrent SHPT (14, 15). PTGs causing persistent or recurrent SHPT are usually refractory to medical treatment and require re-PTx (15). However, re-PTx in the neck or mediastinum area can lead to surgical complications, including recurrent laryngeal nerve injury and postoperative bleeding because of the adhesion due to the initial PTx (16). Several methods have been investigated to prevent re-PTx in the neck or mediastinum area, including the efficacy of preoperative imaging studies, intraoperative parathyroid hormone (PTH) monitoring, and frozen section diagnosis (17–21). However, it is important to note that graft-dependent recurrent SHPT can still occur as autografted parathyroid fragments can be stimulated under chronic kidney disease conditions. Although the incidence of graft-dependent recurrent SHPT is low (only 5%) and causative PTGs in the forearm can easily be removed under local anesthesia (22), preventive measures should still be taken, because re-PTx for graft-dependent recurrent SHPT can be burdensome for patients. Therefore, equal attention should be given to investigating preventative measures for graft-dependent recurrent SHPT and persistent or recurrent SHPT in the neck or mediastinal area. However, few studies have investigated the factors contributing to graft-dependent recurrent SHPT (22, 23). Previously identified contributing factors were based solely on pathological findings (23). However, the results of pathological findings are obtained after the operation and cannot be used to select optimal PTGs for autograft during operation. A thorough understanding of contributing factors to prevent graft-dependent recurrent SHPT, especially those that occur before initial PTx completion, may enable the selection of optimal PTGs from the resected PTGs and help prevent graft-dependent recurrent SHPT. This study aimed to investigate the factors contributing to graft-dependent recurrent SHPT before the completion of initial PTx.

## 2 Materials and methods

### 2.1 Study design

This retrospective cohort study was approved by the Japanese Red Cross Aichi Medical Center Nagoya Daini Hospital Institutional Review Board (approval number: 1577; Aichi, Japan). Factors contributing to graft-dependent recurrent SHPT before initial PTx completion were investigated. The study was conducted in accordance with the principles of the Declaration of Helsinki and Strengthening the Reporting of Observational Studies in Epidemiology (STROBE) guidelines.

### 2.2 Participants

Patients who underwent successful initial total PTx and transcervical thymectomy and received autografted parathyroid fragments derived from only one of the resected PTGs at our center between January 2001 and December 2022 were included in this retrospective cohort study (*n* = 770). To minimize the impact of persistent or recurrent SHPT due to PTGs in the neck or mediastinum, only those patients who had a successful initial PTx were selected. Patients were followed up at 1, 3, 6, and 12 months and annually after PTx. All patient data were collected retrospectively from medical records and analyzed anonymously; therefore, obtaining informed consent from the participants was not required.

### 2.3 Indications of initial PTx and Re-PTx for SHPT

According to the Japan’s Chronic Kidney Disease-related Mineral and Bone Disorder guidelines (24), the following patients were indicated for initial PTx or re-PTx in cases of SHPT: those with intact PTH levels of ≥ 500 pg/mL; those with uncontrolled levels of serum calcium (> 10.0 mg/dL) or phosphorus (> 6.0 mg/dL) even after medical treatment; or those with SHPT symptoms.

### 2.4 Surgical procedure of initial PTx

All patients underwent total parathyroidectomy, transcervical thymectomy, and forearm autograft. The PTGs that appeared most similar to the normal PTGs were selected for autografts. The PTGs were fragmented into 1 × 1 × 3 mm pieces. Thirty fragmented pieces were autografted into the forearm muscle (25).

### 2.5 Definition of successful initial PTx and hypoparathyroidism

As per the Chronic Kidney Disease-related Mineral and Bone Disorder guidelines in Japan, the target range for the serum intact PTH level is 60–240 pg/mL (19). Therefore, serum intact PTH level of < 60 pg/mL on postoperative day 1 was defined as a successful initial PTx. The validity of this definition has been confirmed in previous reports (17, 18). The incidence of re-PTx due to persistent or recurrent SHPT in the neck or mediastinum was significantly lower in patients with serum intact PTH levels < 60 pg/mL on postoperative day 1 than in those with PTH levels ≥ 60 pg/mL. Hypoparathyroidism was defined as persistent serum intact PTH levels < 60 pg/mL after initial PTx.

### 2.6 Pathological findings of PTGs

Pathologists investigated paraffin sections of resected PTGs, and the following hyperplastic patterns were identified: diffuse hyperplasia, concomitant diffuse and nodular hyperplasia, and nodular hyperplasia (26).

### 2.7 Diagnosis of localization of causative PTGs for Re-PTx

The localization of causative PTGs for re-PTx was diagnosed using computed tomography, ultrasonography, and technetium-99m methoxyisobutylisonitrile scintigraphy for persistent or recurrent SHPT in the neck or mediastinum and using ultrasonography and magnetic resonance imaging for recurrent SHPT in the autografted forearm (19, 25).

#### 2.8 Surgical approach for Re-PTx in graft-dependent recurrent SHPT

Re-PTx was performed under local anesthesia, and the autografted PTGs in the forearm muscle were removed with the surrounding muscle to prevent the recurrence of the remaining small PTG fragments (25).

### 2.9 Statistical analysis

Categorical variables were analyzed using the chi-squared test or Fisher’s exact test, whereas continuous variables were analyzed using the Mann–Whitney U test. Cox regression analysis was performed to investigate the factors contributing to re-PTx due to graft-dependent recurrent SHPT and to the prevention of hypoparathyroidism after initial PTx. The analysis included all the following covariates: sex, age, height, body weight, body mass index, diabetic nephropathy, dialysis vintage, preoperative vitamin D receptor activation therapy, preoperative calcimimetic therapy, preoperative serum alkaline phosphatase level, preoperative serum calcium level corrected by serum albumin level, preoperative phosphorus level, serum intact PTH level at admission, maximum diameter and weight of PTG for autograft, and the number of fragmented parathyroid pieces for autograft.

To investigate the impact of the hyperplastic pattern of the PTGs on graft-dependent recurrent SHPT, Cox regression analysis adjusted for dialysis vintage, the maximum diameter of PTG for autograft, and the weight of PTG for autograft was performed. To investigate the impact of the hyperplastic pattern of PTGs on contributing factors to prevent hypoparathyroidism after PTx, Cox regression analysis adjusted for dialysis vintage, maximum diameter and weight of PTG for autograft, and preoperative vitamin D receptor activation therapy was performed. Receiver operating characteristic (ROC) curve analysis was performed to obtain the optimal maximum diameter of PTG for autograft. Statistical analyses were performed using IBM SPSS^®^ Statistics for Windows (version 23.0; IBM Corp., Armonk, NY, USA) and R version 4.0.2 (R Core Team (2020)). Statistical significance was set at *P* value < 0.05 for all analyses.

## 3 Results

### 3.1 Study population

**Figure 1:**
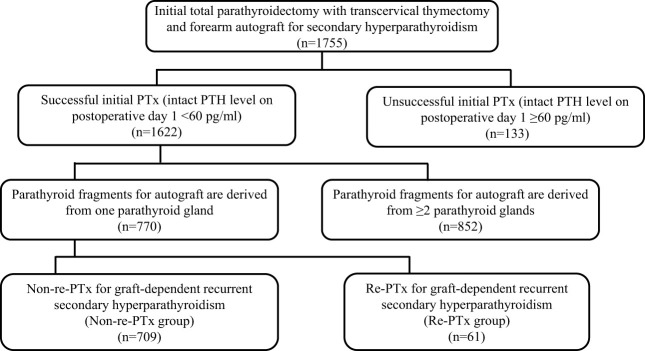
Patient flow chart.

Between January 2001 and December 2022, a total of 1,755 procedures involving PTx, transcervical thymectomy, and forearm autograft were performed. Of these, successful PTx was performed in 1,622 patients (92.4%), while it was unsuccessful in 133 patients (7.6%). Among the 1,622 patients with successful PTx, 770 had parathyroid fragments autografted from only one PTG, while the remaining 852 had parathyroid fragments autografted from ≥ 2 PTGs. The median observation period for the patients included in the final analysis (*n* = 770) was 47.0 months (interquartile range, 17.0–89.0). The patients were classified into two groups based on re-PTx or non-re-PTx for graft-dependent recurrent SHPT (**Figure 1**).

### 3.2 Patient characteristics

With respect to patient characteristics, no significant differences were identified between the two groups, except for dialysis vintage (*P* = 0.003), preoperative serum calcium level (*P* = 0.008), preoperative serum calcium level corrected by serum albumin level (*P* = 0.003), preoperative serum phosphorus level (*P* = 0.009), and serum intact PTH level at admission (*P* = 0.027) (**Table 1**).

**Table 1:** Patient characteristics.

	Non-re-PTx group *n* = 709	Re-PTx group *n* = 61	*P* value	OR	95% CI	
Sex (male), *n* (%)	403 (56.8)	34 (55.7)	0.867	0.956	0.565	1.619
Age (years), mean (SD)	55.3 (11.4)	54.0 (12.3)	0.450			
Height (cm), mean (SD)	160.8 (9.5)	161.8 (10.4)	0.495			
Body weight (kg), mean (SD)	58.0 (13.1)	57.7 (11.2)	0.727			
Body mass index (kg/m^2^), mean (SD)	22.3 (3.7)	22.0 (3.2)	0.756			
Dialysis vintage (months), mean (SD)	145.5 (96.8)	111.2 (48.6)	**0.003**			
Diabetic nephropathy, *n* (%)	78 (11.0)	3 (4.9)	0.190	0.418	0.128	1.367
Preoperative vitamin D receptor activation therapy, *n* (%)	533 (75.2)	42 (68.9)	0.116	0.635	0.359	1.123
Preoperative calcimimetic therapy, *n* (%)	216 (30.5)	14 (23.0)	0.245	0.678	0.366	1.259
Preoperative serum albumin level (g/dL), mean (SD)	3.6 (0.5)	3.5 (0.5)	0.082			
Preoperative serum alkaline phosphatase level (U/L), mean (SD)	451.3 (428.4)	447.0 (357.8)	0.874			
Preoperative serum calcium level (mg/dL), mean (SD)	10.0 (0.8)	10.2 (0.6)	**0.008**			
Preoperative serum calcium level corrected by serum albumin level (mg/dL), mean (SD)	10.4 (0.9)	10.8 (0.8)	**0.003**			
Preoperative serum phosphorus level (mg/dL), mean (SD)	6.2 (4.5)	6.6 (1.6)	**0.009**			
Serum intact PTH level at admission (pg/mL), mean (SD)	882.3 (559.9)	998.9 (515.2)	**0.027**			

CI, confidence interval; OR, odds ratio; PTH, parathyroid hormone; PTx, parathyroidectomy; SD, standard deviation.

Bold font indicates statistically significant results.

### 3.3 Intraoperative and postoperative findings

Significant differences were identified between the two groups with respect to the maximum diameter (*P* = 0.001) and weight (*P* < 0.001) of the PTG for autograft (**Table 2**). Significant differences were identified between the two groups in terms of several variables. These included serum phosphorus levels on postoperative day 1 (*P* = 0.028), serum intact PTH levels on postoperative day 1 (*P* = 0.012), incidence of hypoparathyroidism after initial PTx (*P* < 0.001), and observation period (*P* = 0.003) (**Table 2**). Although postoperative management, including calcium supplementation therapy, vitamin D receptor activation therapy, and kidney transplantation after PTx, was similar in both groups, a higher frequency of postoperative calcimimetics therapy was observed in the re-PTx group (*P* < 0.001).

**Table 2:** Intraoperative and postoperative findings.

		Non-re-PTx group *n* = 709	Re-PTx group *n* = 61	*P* value	OR	95% CI	
Intraoperative results	Maximum diameter of PTG for autograft (mm), mean (SD)	11.8 (3.4)	13.2 (3.5)	0.001			
	Weight of PTG for autograft (mg), mean (SD)	333.8 (266.0)	414.9 (241.4)	< 0.001			
	Number of fragmented parathyroid pieces for autograft, mean (SD)	29.2 (2.7)	29.7 (1.9)	0.087			
Postoperative results	Serum albumin level on POD 1 (g/dL), mean (SD)	3.6 (0.5)	3.5 (0.5)	0.131			
	Serum alkaline phosphatase level on POD 1 (U/L), mean (SD)	451.3 (428.4)	447.0 (357.8)	0.687			
	Serum calcium level on POD 1 (mg/dL), mean (SD)	10.0 (0.8)	10.2 (0.6)	0.726			
	Serum calcium level corrected by serum albumin level on POD 1 (mg/dL), mean (SD)	10.4 (0.9)	10.8 (0.8)	0.832			
	Serum phosphorus level on POD 1 (mg/dL), mean (SD)	6.2 (4.5)	6.6 (1.6)	0.028			
	Serum intact PTH levels on POD 1 (pg/mL), mean (SD)	14.2 (10.4)	19.3 (14.3)	0.012			
	Hyperplastic pattern of PTG			0.348			
	Diffuse hyperplasia, *n* (%)	76 (96.2)	3 (3.8)				
	Diffuse and nodular hyperplasia, *n* (%)	350 (91.4)	33 (8.6)				
	Nodular hyperplasia, *n* (%)	269 (92.1)	23 (7.9)				
	Thyroid papillary carcinoma diagnosed in the paraffin section, *n* (%)	35 (4.9)	4 (6.5)	0.580	1.351	0.464	3.937
	Parathyroid carcinoma diagnosed in the paraffin section, *n* (%)	0	0	NA			
	Serum intact PTH levels before re-PTx for graft-dependent SHPT (pg/mL), mean (SD)		459.4 (276.0)	NA			
	Serum intact PTH levels 1 day after re-PTx for graft-dependent SHPT (pg/mL), mean (SD)		132.7 (160.2)	NA			
	Re-PTx for recurrent SHPT in the neck or mediastinum, *n* (%)	10 (1.4%)	3 (4.9)	0.076			
	Postoperative calcium supplementation therapy, *n* (%)	709 (100.0)	61 (100.0)	> 0.999	NA	NA	NA
	Postoperative vitamin D receptor activation therapy, *n* (%)	709 (100.0)	61 (100.0)	> 0.999	NA	NA	NA
	Postoperative calcimimetics therapy, *n* (%)	52 (8.0)	30 (50.0)	< 0.001	11.558	6.471	20.642
	Kidney transplantation after initial PTx, *n* (%)	33 (5.0)	3 (5.0)	> 0.999	1.000	0.297	3.362
	Hypoparathyroidism after initial PTx, *n* (%)	331 (46.7)	0	< 0.001			
	Death during the observation period, *n* (%)	31 (4.4)	3 (4.9)	0.746	1.130	0.335	3.807
	Observation period (months), mean (SD)	58.7 (53.5)	68.3 (33.9)	0.003			

CI, confidence interval; NA, not accessed; OR, odds ratio; POD 1, postoperative day 1; PTGs, parathyroid glands; PTH, parathyroid hormone; PTx, parathyroidectomy; SD, standard deviation; SHPT, secondary hyperparathyroidism. Bold font indicates statistically significant results.

### 3.4 Factors contributing to Re-PTx due to graft-dependent recurrent SHPT before the completion of the operation

Univariate Cox regression analysis of the factors contributing to re-PTx for graft-dependent SHPT before the completion of operation demonstrated significant differences in dialysis vintage (*P* = 0.007; hazard ratio [HR], 0.995; 95% confidence interval [CI], 0.991–0.999), maximum diameter of PTG for autograft (*P* = 0.002; HR, 1.119; 95% CI, 1.041–1.202), and weight of PTG for autograft (*P* = 0.024; HR, 1.001; 95% CI, 1.000–1.002) (**Table 3**). Multivariate Cox regression analysis demonstrated significant differences in dialysis vintage (*P* = 0.010; HR, 0.995; 95% CI, 0.992–0.999) and the maximum diameter of PTG for autograft (*P* = 0.046; HR, 1.107; 95% CI, 1.002–1.224) (**Table 4**).

**Table 3:** Univariate Cox regression analysis for re-parathyroidectomy due to graft-dependent recurrent secondary hyperparathyroidism.

	*P* value	HR	95% CI	
Sex (male vs. female)	0.717	0.910	0.549	1.511
Age (years)	0.255	1.014	0.990	1.038
Height (cm)	0.924	1.001	0.974	1.030
Body weight (kg)	0.952	0.999	0.978	1.021
Body mass index (kg/m^2^)	0.994	1.000	0.928	1.079
Diabetic nephropathy (vs non-diabetic nephropathy)	0.398	0.606	0.189	1.938
Dialysis vintage (months)	**0.007**	0.995	0.991	0.999
Preoperative vitamin D receptor activation therapy (vs non-preoperative vitamin D receptor activation therapy)	0.076	0.612	0.356	1.052
Preoperative calcimimetic therapy (vs non-preoperative calcimimetic therapy)	0.315	1.366	0.744	2.508
Preoperative serum alkaline phosphatase level (U/L)	0.949	1.000	0.999	1.001
Preoperative serum calcium level corrected by serum albumin level (mg/dL)	0.187	1.229	0.905	1.669
Preoperative serum phosphorus level (mg/dL)	0.481	1.012	0.979	1.047
Serum intact PTH levels at admission (pg/mL)	0.096	1.000	1.000	1.001
Maximum diameter of PTG for autograft (mm)	0.002	1.119	1.041	1.202
Weight of PTG for autograft (mg)	0.024	1.001	1.000	1.002
Number of fragmented parathyroid pieces for autograft	0.938	0.994	0.862	1.147

CI, confidence interval; HR, hazard ratio; PTG, parathyroid gland; PTH, parathyroid hormone. Bold font indicates statistically significant results.

**Table 4:** Multivariate Cox regression analysis for re-parathyroidectomy due to graft-dependent recurrent secondary hyperparathyroidism.

	*P* value	HR	95% CI	
Dialysis vintage (months)	**0.010**	0.995	0.992	0.999
Maximum diameter of PTG for autograft (mm)	**0.046**	1.107	1.002	1.224
Weight of PTG for autograft (mg)	0.832	1.000	0.999	1.001

CI, confidence interval; HR, hazard ratio; PTG, parathyroid gland. Bold font indicates statistically significant results.

### 3.5 Impact of hyperplastic pattern on Re-PTx due to graft-dependent recurrent SHPT

No significant differences were identified regarding the impact of the hyperplastic pattern of autografted PTGs on re-PTx for graft-dependent recurrent SHPT both in unadjusted (*P* = 0.568) and adjusted Cox regression analyses for dialysis vintage and in maximum diameter and weight of PTG (*P* = 0.732) (**Table 5**).

**Table 5:** Impact of hyperplastic pattern on re-parathyroidectomy due to graft-dependent recurrent secondary hyperparathyroidism.

	Re-PTx group	Non-re-PTx group	Unadjusted				Adjusted for dialysis vintage, maximum diameter of PTG, and weight of PTG			
	*n* = 59	*n* = 695	HR	95% CI		*P* value	HR	95% CI		*P* value
Diffuse hyperplasia, *n* (%)	3 (3.8)	76 (96.2)	ref			0.568	ref			0.732
Diffuse and nodular hyperplasia, *n* (%)	33 (8.6)	350 (91.4)	1.900	0.582	6.204	0.288	1.507	0.453	5.014	0.504
Nodular hyperplasia, *n* (%)	23 (7.9)	269 (92.1)	1.817	0.544	6.062	0.331	1.634	0.480	5.566	0.432

CI, confidence interval; HR, hazard ratio; PTG, parathyroid gland; PTx, parathyroidectomy; ref, reference.

### 3.6 Factors contributing to the prevention of hypoparathyroidism after initial PTx

Univariate Cox regression analysis of the factors contributing to the prevention of hypoparathyroidism after PTx showed significant differences in dialysis vintage (*P* < 0.001; HR, 0.996; 95% CI, 0.995–0.998), preoperative vitamin D receptor activation therapy (*P* = 0.008; HR 0.748; 95% CI, 0.603–0.928), serum intact PTH levels at admission (*P* = 0.009; HR, 1.000; 95% CI, 1.000–1.000), maximum diameter of PTG for autograft (*P* = 0.019; HR, 1.033; 95% CI, 1.005–1.061), and weight of PTG for autograft (*P* = 0.028; HR, 1.000; 95% CI, 1.000–1.001) (**Table 6**). Multivariate Cox regression analysis demonstrated significant differences in dialysis vintage (*P* < 0.001; HR, 0.996; 95% CI, 0.995–0.998). However, no significant differences were identified in the maximum diameter (*P* = 0.474; HR, 1.015; 95% CI, 0.975–1.056) and weight of PTG for autograft (*P* = 0.586; HR, 1.000; 95% CI, 1.000–1.001) (**Table 7**).

**Table 6:** Univariate Cox regression analysis for the factors contributing to the prevention of hypoparathyroidism after initial parathyroidectomy.

	*P* value	HR	95% CI	
Sex (male vs. female)	0.077	0.844	0.699	1.019
Age (years)	0.658	1.002	0.994	1.010
Height (cm)	0.110	0.992	0.982	1.002
Body weight (kg)	0.288	0.996	0.988	1.004
Body mass index (kg/m^2^)	0.785	0.996	0.971	1.023
Diabetic nephropathy (vs non-diabetic nephropathy)	0.651	0.927	0.667	1.288
Dialysis vintage (months)	**< 0.001**	0.996	0.995	0.998
Preoperative vitamin D receptor activation therapy (vs non-preoperative vitamin D receptor activation therapy)	**0.008**	0.748	0.603	0.928
Preoperative calcimimetic therapy (vs non-preoperative calcimimetic therapy)	0.925	1.010	0.820	1.244
Preoperative serum alkaline phosphatase level (U/L)	0.066	1.000	1.000	1.000
Preoperative serum calcium level corrected by serum albumin level (mg/dL)	0.973	0.998	0.898	1.109
Preoperative serum phosphorus level (mg/dL)	0.960	1.000	0.984	1.017
Serum intact PTH levels at admission (pg/mL)	**0.009**	1.000	1.000	1.000
Maximum diameter of PTG for autograft (mm)	**0.019**	1.033	1.005	1.061
Weight of PTG for autograft (mg)	**0.028**	1.000	1.000	1.001
Number of fragmented parathyroid pieces for autograft	0.261	0.979	0.943	1.016

CI, confidence interval; HR, hazard ratio; PTG, parathyroid gland; PTH, parathyroid hormone. Bold font indicates statistically significant results.

**Table 7:** Multivariate Cox regression analysis for the factors contributing to the prevention of hypoparathyroidism after initial parathyroidectomy.

	*P* value	HR	95% CI	
Dialysis vintage (months)	**< 0.001**	0.996	0.995	0.998
Maximum diameter of PTG for autograft (mm)	0.474	1.015	0.975	1.056
Weight of PTG for autograft (mg)	0.586	1.000	1.000	1.001
Serum intact PTH levels at admission (pg/mL)	0.055	1.000	1.000	1.000

CI, confidence interval; HR, hazard ratio; PTG, parathyroid gland. Bold font indicates statistically significant results.

### 3.7 Impact of hyperplastic pattern on the prevention of hypoparathyroidism after initial PTx

No significant differences were identified regarding the impact of the hyperplastic pattern of autografted PTGs on the prevention of hypoparathyroidism after initial PTx, both in the unadjusted (*P* = 0.163) and adjusted Cox regression analyses for dialysis vintage, maximum diameter of PTG, and weight of PTG, as well as preoperative vitamin D receptor activation therapy (*P* = 0.485) (**Table 8**).

**Table 8:** Impact of hyperplastic pattern on the prevention of hypoparathyroidism after initial parathyroidectomy.

	Hypoparathyroidism after initial PTx	Non-hypoparathyroidism after initial PTx	Unadjusted				Adjusted for dialysis vintage, maximum diameter of PTG, weight of PTG, and preoperative vitamin D receptor activation therapy			
	*n* = 324	*n* = 429	HR	95% CI		*P* value	HR	95% CI		*P* value
Diffuse hyperplasia, *n* (%)	33 (10.2)	46 (10.7)	ref			0.163	ref			0.485
Diffuse and nodular hyperplasia, *n* (%)	151 (46.6)	232 (54.1)	1.059	0.771	1.453	0.724	1.065	0.766	1.481	0.709
Nodular hyperplasia, *n* (%)	140 (43.2)	151 (35.2)	0.868	0.623	1.207	0.399	0.936	0.661	1.324	0.708

CI, confidence interval; HR, hazard ratio; PTG, parathyroid gland; PTx, parathyroidectomy; ref, reference.

### 3.8 ROC curve analysis for the optimal maximum diameter of PTG for autograft

**Figure 2:**
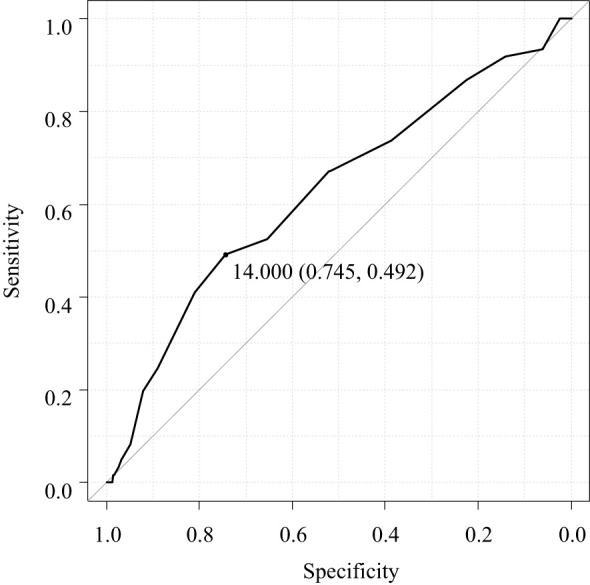
Receiver operating characteristic curve analysis for the optimal maximum diameter of the parathyroid gland for autograft.

ROC curve analysis for the optimal maximum diameter of PTG for autograft demonstrated that a maximum diameter of < 14.0 mm was the optimal cutoff to prevent re-PTx for graft-dependent SHPT (area under the curve, 0.628; 95% CI, 0.551–0.705) (**Figure 2**).

## 4 Discussion

To the best of our knowledge, this is the first study to investigate the factors contributing to re-PTx due to graft-dependent recurrent SHPT before the completion of initial PTx and prevention of hypoparathyroidism after initial PTx. The results showed that dialysis vintage and the maximum diameter of the PTG for autograft were significant contributors to re-PTx due to graft-dependent recurrent SHPT. However, the maximum diameter of the PTG for autograft was not a significant contributor toward preventing hypoparathyroidism after PTx. Furthermore, the pathological hyperplastic pattern of PTGs did not contribute to the re-PTx or prevention of hypoparathyroidism after initial PTx. The optimal maximum diameter of PTG for autograft to prevent graft-dependent recurrent SHPT was < 14.0 mm.

A previous study reported that PTG autografts with nodular hyperplastic patterns are associated with a higher probability of re-PTx than those with diffuse hyperplastic patterns (23). A maximum PTG diameter of ≥ 8 mm during preoperative ultrasonography is reported to be associated with a nodular hyperplastic pattern of PTGs (27). Thus, PTGs measuring ≥ 8 mm in diameter during preoperative ultrasonography should not be used for autografts. However, it should be noted that preoperative imaging studies may not accurately diagnose PTGs, as they may misdiagnose lymph nodes or parts of the thyroid gland as PTGs, and undetected PTGs are also common (19, 28). Therefore, PTGs for autografts should be selected during the surgery itself, although the hyperplastic pattern cannot be diagnosed during the short duration of the surgery. The present study investigated factors contributing to re-PTx due to graft-dependent SHPT in patients with autografted parathyroid fragments derived from only one resected PTG. These patients had successful initial total PTx and transcervical thymectomy, as defined by serum intact PTH level < 60 pg/mL on postoperative day 1. To better understand the impact of patient and autografted PTG characteristics, only patients who were autografted from one of the resected PTGs were selected. Furthermore, only patients with successful initial total PTx and transcervical thymectomy were selected to minimize the impact of persistent or recurrent SHPT in the neck or mediastinal area. Previously, serum intact PTH levels < 60 pg/mL on postoperative day 1 were demonstrated to be a significant predictive factor of successful PTx, although it has less than 100% accuracy, because persistent or recurrent SHPT in the neck or mediastinal area was identified in 13 (1.7%) patients, similar to previous reports (17, 18). Nonetheless, the incidence of persistent or recurrent SHPT was still markedly lower compared with that of other reports (5–30%), indicating the usefulness of postoperative serum intact PTH level as a predictor of SHPT persistence or recurrence (19, 29–31).

Our results indicate that the maximum diameter of the PTG for autograft is a significant factor that can cause re-PTx due to graft-dependent SHPT, while the weight of the PTG was not a causative factor. A previous study reported that nodular hyperplasia in autografted PTGs could be associated with re-PTx (23). The present study investigated the impact of hyperplastic patterns on the re-PTx due to graft-dependent SHPT with adjustment for patients and PTG characteristics obtained before the operation’s completion. However, in contrast to previously published data, our results did not reveal any correlation of the hyperplastic pattern with the incidence of re-PTx. One possible explanation for these disparate findings could be that in the previous study, the patients and PTG characteristics were not adjusted using multivariate analysis, and the hyperplastic patterns were only categorized into diffuse and nodular hyperplasia. Another study reported a progressive change in hyperplastic pattern from diffuse to nodular hyperplasia and the concomitant existence of both types of hyperplasia (26). In contrast, the current study classified hyperplastic patterns into three distinct categories: diffuse hyperplasia, concomitant diffuse and nodular hyperplasia, and nodular hyperplasia. Concomitant diffuse and nodular hyperplasia was identified in 50.7% (383 out of 754) of PTGs. However, it should be noted that the selection of PTGs for autograft was based on their similarity to the normal PTGs. This selection bias may contribute to the similar results observed in hyperplastic patterns for re-PTx. To completely eliminate this bias, a randomized clinical trial would be necessary. To investigate the PTG characteristics associated with graft-dependent SHPT, this study only included patients who received autografted parathyroid fragments from a single resected PTG. Our results indicate that PTGs selected for autografts should be smaller than a certain diameter. ROC curve analysis demonstrated that PTGs with a maximum diameter < 14 mm might be the most suitable for autografts.

The present study investigated the prevention of hypoparathyroidism after initial PTx, as it has serious consequences such as low bone turnover and ectopic calcification, which can lead to cardiac events (32, 33). However, except for the duration of dialysis treatment, no other significant factors contributing to the prevention of hypoparathyroidism were identified among the characteristics of PTGs examined. Previously, it has been reported that intact PTH levels might increase with an increase in the autografted number of PTGs following total thyroidectomy (34). Herein, 30 fragmented parathyroid pieces were autografted; however, the effect of the number of autografted parathyroid pieces on hypoparathyroidism after initial PTx could not be investigated. Additionally, the hyperplastic pattern of PTGs did not affect the incidence of hypoparathyroidism after initial PTx, suggesting that the hyperplastic pattern might not affect the function of the autografted parathyroid pieces.

The study has some limitations including its retrospective nature. Further, the impact of the number of autografted parathyroid pieces on hypoparathyroidism after initial PTx could not be investigated. The selection bias of PTGs for autograft could not be completely eliminated in this study, and future studies should adopt a prospective randomized approach to investigate the impact of autografted PTGs, including the number of autografted parathyroid pieces, on re-PTx and hypoparathyroidism.

In conclusion, the maximum diameter of the PTG for autograft is the factor contributing to re-PTx due to graft-dependent recurrent SHPT. PTGs with a maximum diameter of < 14 mm may be appropriate for autografts. These results may help surgeons to select optimal PTGs for autografts to prevent graft-dependent SHPT.

## Data availability statement

The raw data supporting the conclusions of this article will be made available by the authors, without undue reservation.

## Ethics statement

The studies involving human participants were reviewed and approved by Japanese Red Cross Aichi Medical Center Nagoya Daini Hospital Institutional Review Board. Written informed consent for participation was not required for this study in accordance with the national legislation and the institutional requirements.

## Author contributions

TH designed and acquired the data, interpreted the results, and drafted the manuscript; MO acquired the data; YH, KF, NG, YT, SN, YW, and TI interpreted the results. All authors contributed to the article and approved the submitted version.
